# Gardette’s Proposition to Establish a Lectureship on Dental Surgery in Medical Colleges

**Published:** 1852-01

**Authors:** E. B. Gardette


### Editorial.

### PROPOSITION TO ESTABLISH A LECTURESHIP ON DENTAL SURGERY IN MEDICAL COLLEGES.

BY E. B. GARDETTE.

"The undersigned, a practicing dentist of Philadelphia, begs leave respectfully to call the attention of the trustees and medical faculties of the medical schools of the United States to the propriety and advantages of establishing an adjunct professorship to the chair of surgery, in which the specialty of dental surgery may be taught to the medical student seeking knowledge in your institutions.

"In making this suggestion, he indulges the confident belief that the existence of such a chair would be no less useful to those who may be compelled to practice some branch of dental surgery, as part of their duties in general surgery, than to the smaller number who may determine to embrace that specialty as their profession.

"The undersigned would offer for your consideration some reflections that seem to render this proposal consistent, not only with the wants of the student of medicine and the public, but with the duty and the interests of the medical schools which may act upon this request in such form as seems most proper to their own judgment.

"It will scarcely be assumed by any trustee, and still less by any member of a medical faculty, that the profession of the dentist or its duties, are less important to mankind than any other of the specialties of surgery—whether the oculist, the aurist, or the lithotomist; and therefore it is needless to consider such a question open for debate, or requiring argument in a country, especially, where the services of good dentists have been so widely and universally needed. It being granted, then, that the science itself is of equal importance with the other departments of surgery, it is an undeniable truth that, whilst all other specialties are taught and included in the professorships of medical schools generally, the principles and practice of dental surgery have received no attention, or certainly none that would permit the graduate of medicine to feel that he was qualified for the simplest duty of the dentist.

"Such a state of things was perhaps an unavoidable evil when men could not be found who were competent to teach the principles of our science, and when there was need of a comparatively small number of dentists; and hence no such aid was then called for from the medical colleges; but at this time there would seem to be required about twenty-five hundred dentists, more or less, throughout the United States, which is sufficiently proved by the fact that about that number are believed to be engaged in the practice of the profession, after some fashion or other--good, bad, and indifferent; and to supply an increasing demand for well-educated dental surgeons, as well as to raise the standard of that profession, would appear to be a matter of common interest.

"It would seem an act of inexplicable unkindness to have thus singled out the profession of dentist from among the specialties of surgery, and forced it into an attitude of independent self-protection; but a gentleman who fulfils the duties of both medical practitioner and dentist with such distinguished abilities as to be an honor to both professions, has traced truthfully the origin of the neglect of which the dentist complains. The undersigned quotes from a valedictory address to the graduating class of Baltimore College of Dental Surgery, March, 1850, when speaking of the early periods of the existence of a medical faculty:

"'They condemned, without stint, a calling they knew not how to practice, and a practice they knew not how to improve. Such of the faculty as were learned in their profession were found always competent and fully prepared to be oculists, aurists, or lithotomists, or to devote themselves to any other branch of the profession which their interest, inclination, or talents might determine, *except* that of dental surgery. This branch seemed to require something more than medical knowledge; it required great mechanical skill—an education of the hand as well as of the head—a kind of education they had not received and knew not where to acquire, and yet affected to despise. The necessities of the community cried aloud to them for help—a help which they could not bestow. This drove many sufferers to seek dental aid out of the medical profession, and to obtain that help which mechanical genius alone could supply. At this the profession seemed mortified and chagrined, and loudly mocked at those who dared to supply their delinquencies, and united as one man in deriding the uneducated dental mechanic. They first created the necessity for an empiric, and then croaked forth their withering contempt on the creature their own ignorance had made.'

"Thus has dental surgery been left to struggle through endless impediments and difficulties, instead of being regarded as a link, however humble, in the great chain of medical science, which should with patriarchal strength and harmony foster and embrace every branch of the healing art.

"It is now believed that, among the number of medical students who attend each of the schools, there are many who would gladly devote themselves to the specialty of dental surgery, and it is from among these, and at the early age at which they generally devote themselves to medical studies, that good dentists could be formed; they should still be required to earn and receive a diploma with the title of M. D., as a guarantee of fitness to practice and a claim to confidence as dentist. Even among the number that annually graduate, either to practice medicine generally, or limiting their attendance to their own families and dependants at the South, a correct knowledge of the theory and practice of dental surgery, such as might be derived from a course of lectures, would be highly valuable. And it is believed there would not only be a direct increase of good dentists, but a desirable addition to the number of those who should be competent to determine what constitutes a good dentist.

"The establishment of separate schools for each specialty in medical science (and why should there not be for the oculist, or the aurist, as well as the dentist?) would appear to be the dismembering of a great family, thereby lessening its influence for good, as well as that power which is justly derived from an *esprit de corps* subsisting in every branch of an elevated and honorable profession. In this expression of opinion as to the ultimate tendency of dental colleges, the undersigned is actuated by no unkind or invidious feelings, but, on the contrary, he gives them due credit for the efforts they have made and are making to improve the profession; and to the Baltimore College of Dental Surgery he is personally grateful for acts of courtesy and kindness towards himself, and to the memory of his father.

"The educated dentists in the various parts of the United States are abundantly numerous, it is believed, to fulfil such duties as might be assigned them in each of our medical colleges. The students who now attend the dental schools would, in this event, be added to the number of matriculants in the medical classes of the country, and the need of separate institutions would cease.

"Frequent occasion for consultations between the physician, the surgeon, and the dentist, has been felt by almost every practitioner of medicine, as a necessary consequence of the connection and sympathy reciprocally existing between the teeth and many serious disturbances of the general health: such sympathies would naturally seem to suggest the mutual dependence between the physician, the surgeon, and the dentist, and should be a good reason for some similarity and sympathy in their modes of education. The benefits from such a state of things as the mind can readily anticipate would not rest here; medical men would sometimes, no doubt, receive important aid from the dentist, in tracing to their true origin many diseases of the head and face that now baffle their skill.

"Whatever action your medical faculty or board of trustees may see fit to take in reference to these suggestions, and the object they are designed to promote, the undersigned may at least respectfully urge that they would seem to demand fair and kindly consideration, as involving matters of deep concern to the community, for whose safety and advantage all medical learning is sought, and medical colleges instituted and fostered. E. B. GARDETTE."

—

We give place to the above proposition in the present number of the Register, and fully intended having done so before, particularly in our last issue, but Prof. Harris had so fully answered the proposition, or rather shown the impracticability of such a thing, that we felt as if it scarcely required a notice from us. We are aware, however, that some diversity of opinion exists on this subject, and as it appears to be "*the day*" for dental colleges, we think it important that they should be established on a proper basis. We have the utmost respect for Dr. Gardette as a man and practitioner, and therefore hope that our opinions will not be regarded as anything else than the result of observation and a conviction that their adoption would conduce to the greatest good of the profession, as well as the public. Our first objection to the proposition is, that while it would fail to impart that thorough practical knowledge to the student which is necessary to the dental practitioner, it would give to the medical practitioner just enough of the practical details of the profession to induce him to resort to it as a dernier resort, after he had failed to succeed in that which he had taken special pains to qualify himself for. That this would be the working of the system we think every observing and candid man must admit. There is no possible way to remedy this, unless the term of study for the student of medicine should be prolonged at least one year, and that regardless of the time necessary for the study of mechanical dentistry. This is strictly in accordance with the requisitions of the Ohio College of Dental Surgery. Medical Practitioners are required to study the practical details of dental surgery one year, and attend one course of lectures in the institution before they can graduate; and this is necessary even if he should be a graduate, if not, he should be eligible for graduation in a medical college by one course of lectures—such as a practice of four years, &c. True, we are glad to see regular practitioners of medicine turning their attention to the specialty of dentistry, but not after they have failed to succeed in the former, or after they have passed their period of youth, when their hands are most tractable. If they select dental surgery as the profession of their choice, we wish it to be a choice of taste, not of necessity; and this choice should be made early so that their hands should be early trained to those manipulations requisite in dental practice.

Would medical graduates generally devote that time and attention to dentistry which is necessary, we think such would not be the case if, in their regular medical course, they had heard a few lectures on dental surgery, (and that they could hear more than a few such lectures no one could pretend to say.) A few such lectures we would consider essential to the medical practitioner, yet scarcely what is the beginning of that which is necessary to the dental practitioner.

Such lectures, however, should have more reference to the cause and effect of dental disease, and the therapeutical treatment, than the surgical. This would benefit the medical practitioner, and be of essential service in practice. Operative dentistry could not be properly taught in two or three courses of lectures by the best qualified adjunct professor of dental surgery which could be procured. A very different kind of training is necessary for the dental practitioner.

How the title of M. D. could thus be made evidence of dental qualifications we cannot see, unless indeed all were made good dentists as well as physicians. Yet such appears to be the views of Dr. Gardette, for he remarks that "They (the students) should still be required to earn and receive a diploma with the title of M. D., as a guarantee of fitness to practice, and a claim to confidence as dentist." Here the title of M. D. is made to be essential to dental practice, and with this he is expected to command "*confidence*" as a dentist; and such would be his expectations, and such would be the practical operation of the system, whether the M. D. knew anything of dentistry or not.

We need not be told that the one has made the practical details of dental surgery his special study; his title of M. D. bears no evidence of that, and how should it, for such a course could scarce give such qualification. We are advocates for a thorough medico-dental education, and it was owing to this fact and the great destitution which prevailed in this particular, that induced us to urge the necessity of dental institutions to remedy this evil. Our medical colleges had neglected this branch of surgical science until the remarks quoted by Dr. Gardette from Dr. Hullihan's address, were very just and appropriate. To remedy this evil, for it was an evil, dental institutions were instituted. They had secured the confidence, and, to some considerable extent, the co-operation of the medical profession, and the community at large. They had just passed through a hard struggle for existence and influence, when Dr. Gardette comes with his proposition to supercede them, and for what? That the whole fraternity, medical and dental, may be sheltered under the same form of sheepskin, that the term Doctor of Dental Surgery may be submerged in that of Doctor of Medicine. We confess we like the former, it is significant, and we regard it alike honorable. Another reason we have for dissenting from this proposition is, that it would fail to enlist the dental profession. Not one in a hundred of those now engaged in dental practice could be induced to attend one or two courses of lectures in a medical school and get a diploma. Many such have entered the profession but illy qualified to discharge its duties. They feel at present more need of practical knowledge than theoretical, and was it not for the prospect of gaining this practical knowledge, they would not attend college at all. To gain this, however, they are required to attend all the lectures, and they soon thus feel more deeply the need of that knowledge of anatomy, physiology, pathology, therapeutics, chemistry, &c., which they had regarded as merely useless. We think we are right in asserting that half the present graduates of dental surgery in the United States, are those who had been in actual practice over four years before entering college at all. Many of these have stood as critical examinations in all these branches as graduates of medicine. We therefore expect our dental institutions not only to benefit those now just turning their attention to the study, but those already engaged in the pursuit.

There are many suggestions in relation to this subject which might be dwelt upon in this place, but our present limits forbid, and we have already occupied more space than we intended.

We wish, however, in connection with this subject, to refer to a series of articles by Mr. Wood, Dentist, of Nashville, Tenn., on dental education, one of which is published in the Dental Times for December, 1851. Mr. Wood suggests that to "satisfy the demands of the more ambitious class of dentists, and also meet the approval of medical men generally," the dental student be required to attend "one course of lectures in a medical school previous to his admission or graduation in a dental college." We scarce know whether to regard this as a compliment to dental colleges or not. The inference is, however, that dental schools are not capable of giving proper preparatory instruction. The fact is, that many in the profession appear to be but little acquainted with the course of instruction given in our dental colleges. Or if they are, they have the same feeling with regard to medical schools, which the public formerly had with reference to French dentists. If he hailed from France, and was addressed as *Monsieur*, it was evidence of dental qualification. We rejoice that the profession has outlived this, and hope it may survive the other, and come to realize that she has within herself all that which is necessary to ennoble and sustain herself.

One other remark of Mr. Wood's requires a passing notice. He remarks that "no sooner had the news of the 'failure' of the Ohio College time to spread over the country," &c., &c. Now we do not admit the Ohio College was ever a "failure." That the institution has labored under adverse circumstances, we admit, but not that it has ever proved a failure. Two sessions we had small classes—only seven students each—but some of our oldest medical schools had not as large comparatively to start on. We know that many false statements have been sent out in relation to the Ohio College, but we regard the institution as *successful* over all the opposition and machinations of those who should have been found steadfast sustainers of it. In our next we may allude to the plan proposed by Mr. Wood for the establishment of dental schools. We hope that Dr. Gardette, and also Mr. Wood, will take our remarks in that frank and candid manner in which they are given. We feel that it is principle, and the good of the profession we contend for, and nothing else.

